# Procyanidin A1 Alleviates Inflammatory Response induced by LPS through NF-κB, MAPK, and Nrf2/HO-1 Pathways in RAW264.7 cells

**DOI:** 10.1038/s41598-019-51614-x

**Published:** 2019-10-21

**Authors:** Shan Han, Hongwei Gao, Shaoru Chen, Qinqin Wang, Xinxing Li, Li-Jun Du, Jun Li, Ying-Ying Luo, Jun-Xiu Li, Li-Chun Zhao, Jianfang Feng, Shilin Yang

**Affiliations:** 1 College of Pharmacy, Guangxi University of Chinese Medicine, Nanning, 530000 China; 2 Guangxi Engineering Technology Research Center of Advantage Chinese Patent Drug and Ethnic Drug Development, Nanning, 530020 China; 3 Department of Integrative Medical Sciences, Northeast Ohio Medical University, Rootstown, Ohio 44272 USA; 4 School of Life Sciences, Tsinghua University, Beijing, 100084 China; 5 State Key Laboratory of Innovative Drug and Efficient Energy-Saving Pharmaceutical Equipment, Jiangxi University of Traditional Chinese Medicine, Nanchang, 330004 China

**Keywords:** Molecular medicine, Drug development

## Abstract

Inflammation is a complex physiological process that poses a serious threat to people’s health. However, the potential molecular mechanisms of inflammation are still not clear. Moreover, there is lack of effective anti-inflammatory drugs that meet the clinical requirement. Procyanidin A1 (PCA1) is a monomer component isolated from Procyanidin and shows various pharmacological activities. This study further demonstrated the regulatory role of PCA1 on lipopolysaccharide (LPS)-stimulated inflammatory response and oxidative stress in RAW264.7 cells. Our data showed that PCA1 dramatically attenuated the production of pro-inflammatory cytokines such as NO, iNOS, IL-6, and TNF-α in RAW264.7 cells administrated with LPS. PCA1 blocked IκB-α degradation, inhibited IKKα/β and IκBα phosphorylation, and suppressed nuclear translocation of p65 in RAW264.7 cells induced by LPS. PCA1 also suppressed the phosphorylation of JNK1/2, p38, and ERK1/2 in LPS-stimulated RAW264.7 cells. In addition, PCA1 increased the expression of HO-1, reduced the expression of Keap1, and promoted Nrf2 into the nuclear in LPS-stimulated RAW264.7 cells. Cellular thermal shift assay indicated that PCA1 bond to TLR4. Meanwhile, PCA1 inhibited the production of intracellular ROS and alleviated the depletion of mitochondrial membrane potential *in vitro*. Collectively, our data indicated that PCA1 exhibited a significant anti-inflammatory effect, suggesting that it is a potential agent for the treatment of inflammatory diseases.

## Introduction

A large number of studies have shown that inflammation is a defensive body response after stimulation by all kinds of ligands. However, in some cases, inflammation is potentially harmful. If inflammation cannot be effectively controlled, it will lead to excessive production of pro-inflammatory factors and a series of pathological change, such as diabetes, pneumonia, hepatitis, and nephritis^1,2^. There are overwhelming studies on inflammation recently, among which the most popular *in vitro* model is RAW264.7 cell induced by LPS^3^. RAW264.7, a monocyte/macrophage-like cell line, is also the most commonly used *in vitro* study on screening anti-inflammatory activity from natural compounds. LPS is a common inducer from *Escherichia coli* O111:B4^4,5^. LPS can upregulate a large number of inflammatory mediators such as nitric oxide (NO), cyclooxygenase-2 (COX-2), tumor necrosis factor α (TNF-α), interleukin 6 (IL-6), etc. in RAW264.7 cells during the inflammatory reaction^6^.

As an important transcriptional regulation factor, Nuclear factor (NF-κB) participates in regulating the body of chronic inflammation and produce certain physiological changes^7^. The NF-κB family members including p50, p52, Rel (p65), c-Rel, and RelB, contain one N-terminal Rel homology region that mediates polymerization, DNA binding, and c-terminal nuclear localization sequence^8^. The most abundant and active form of NF-κB is p65-p50 heterodimer. At rest, the IκB protein binds to the nuclear NF-κB subunit and stays in the cytoplasm^9^. Specifically, under stimulation, IKK is activated and leads to degradation of the phosphorylation-dependent proteasome IκB, and then releases NF-κB subunit from the cytoplasm to the nucleus. The NF-κB subunit binds to the κB element and initiates the expression of target genes related to the inflammatory response^10^. MAPKs pathway also plays an important role in the inflammatory process. The family members of MAPKs mainly include extracellular signal-regulated kinase (ERK), stress-activated protein kinase (JNK), and p38 mitogen-activated protein kinase (p38MAPK)^11^. In general, LPS stimulation promotes the phosphorylation of ERK, JNK, and p38 to further stimulate nuclear translocation of the nuclear transfer-factor AP-1and induction of some inflammatory factors expression^11^. Therefore, inhibition of the NF-κB and MAPKs signaling pathway can be a good therapy method on some inflammatory diseases.

Oxidative stress also plays an important role in the inflammation involved in chronic diseases. Nuclear factor erythroid 2-related factor (Nrf2) can regulate the antioxidant proteins expression and protect the body from oxidative damage caused by injury and inflammation^12^. Normally, Nrf2 binds with its suppressor Keap1 in the cytoplasm. While in the activation state, Nrf2 translocates into the nucleus where it binds to ARE and induces the expression of downstream target genes such as HO-1^13^. Therefore, regulation of Nrf2 nucleus translocation and increasing the activity of HO-1 can appropriately inhibit the occurrence of inflammatory reactions^14^.

Procyanidin, a polyphenolic compound existing in most plants, exhibits anti-inflammatory, anti-oxidant, anti-apoptotic and other pharmacological effects^15,16^. Procyanidin A1 (PCA1), as one of the procyanidins that were isolated and identified, drew attention to further research. Previous studies showed that PCA1 has immune modulation, cholesterol modulation, and anti-oxidative effects^17,18^. However, the role of PCA1 in the inflammatory response and oxidative stress is still lack of research evidence. To clarify the role of PCA1 in regulating inflammation more clearly, it is necessary to do further investigation on the anti-inflammatory and anti-oxidative mechanisms of PCA1. Thus, in this study, using LPS-stimulated RAW264.7 cells, we investigated the anti-inflammatory activity and the molecular mechanism of PCA1.

## Results

### PCA1 diminished LPS-stimulated inflammatory reaction in RAW264.7 cells

**Figure 1:**
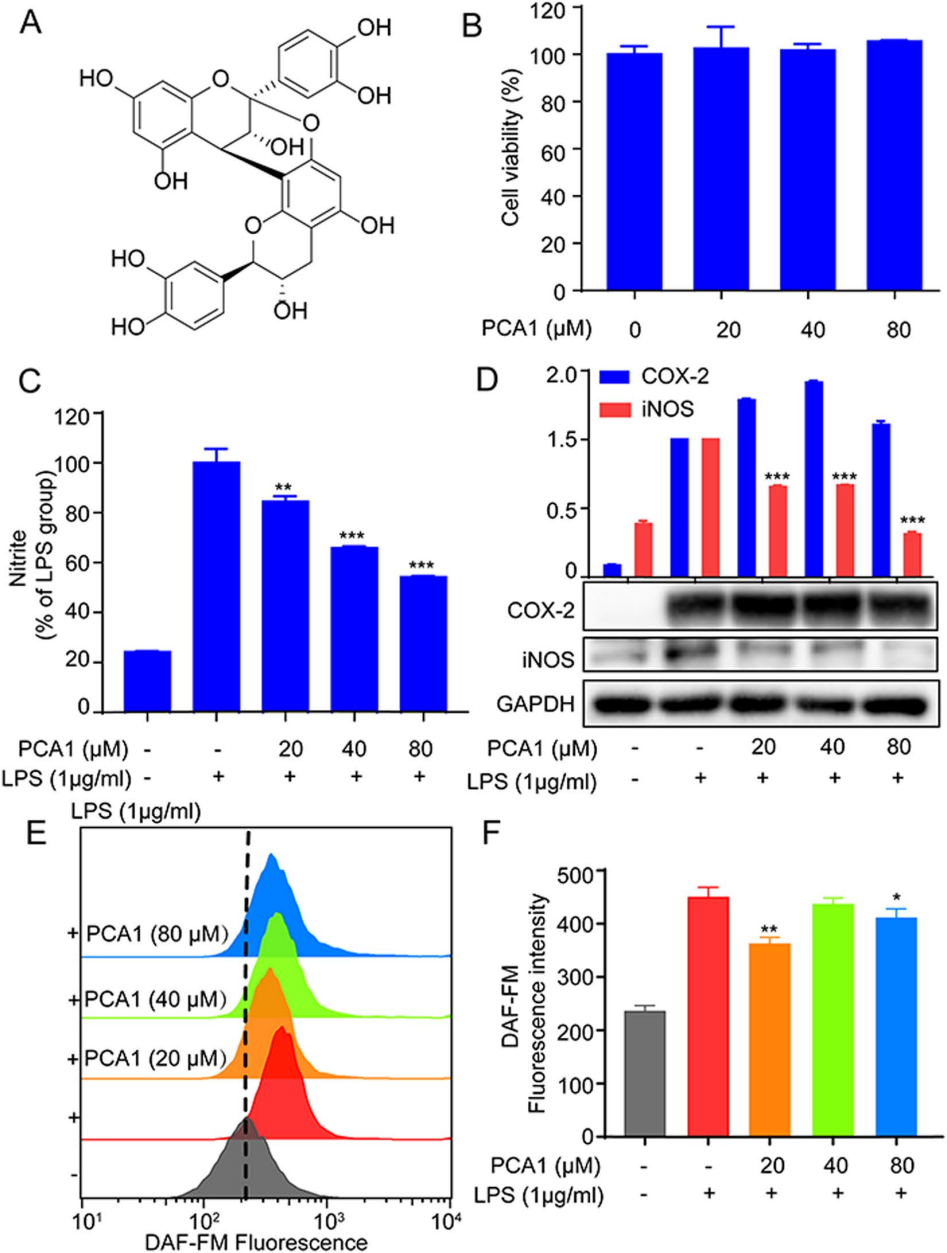
PCA1 diminished LPS-stimulated inflammatory response in RAW264.7 cells. (**A**) Chemical structures of Procyanidin A1 (PCA1). (**B**) The cytotoxicity of PCA1 in RAW264.7 cells was determined by MTT assay after 24 h treatment. (**C**) RAW264.7 cells treated with PCA1 (20, 40, 80 μM) for 1 h and then induced with LPS (1 μg/ml) for 18 h. The medium was collected to determine the nitrite level using Griess Regent. (**D**) RAW264.7 cells treated with PCA1 (20, 40, 80 μM) for 1 h and then induced with LPS (1 μg/ml) for 18 h. iNOS and COX-2 expressions were detected by Western blotting. The grouping of gels/blots cropped from different parts of the same gel (targets vs loading control). The full blots are shown in Supplementary Information. (**E**) RAW264.7 cells were the treatment of PCA1 (20, 40, 80 μM) for 2 h before the co-treatment of LPS (1 μg/ml) for 6 h. Then the cells were marked with DAF-FM for 1 h, which were assessed by flow cytometry. (**F**) Statistical analysis of the NO per group. **p < 0.01, and ***p < 0.001 versus the LPS induced group.

The cytotoxicity of PCA1 (Fig. 1A) was determined by MTT assay. As shown in Fig. 1B, the results indicated that PCA1 (20, 40, 80 μM) displayed no significant toxicity in RAW264.7 cells compared with normal control (NC). Nitrite level, NO release, iNOS, and COX-2 are major indicators of inflammatory response^19^. Our study demonstrated that PCA1 pretreatment suppressed LPS-stimulated nitrite level and iNOS protein expression in a dose-dependent manner. From Fig. 1C, the nitrite level of cells treated with LPS sharply increased. However, the pretreatment with PCA1 in 20 μM exhibits 19.5% inhibition on nitrite level, 37.4% in 40 μM, 47.6% in 80 μM, compared with LPS group (Fig. 1C,D), respectively. In addition, as shown in Fig. 1E,F, the flow cytometry assay indicated that PCA1 diminished NO production in 20 μM. However, we found that PCA1 has no effect on the expression of COX-2 protein (Fig. 1D). As shown in Fig. 1E,F, using the flow cytometry assay, PCA1 suppressed NO release in LPS-stimulated RAW264.7 cells in accordance with the results of the nitrite level and iNOS protein expression.

### PCA1 reduced LPS-induced the expression pro-inflammatory cytokines in RAW264.7 cells

**Figure 2:**
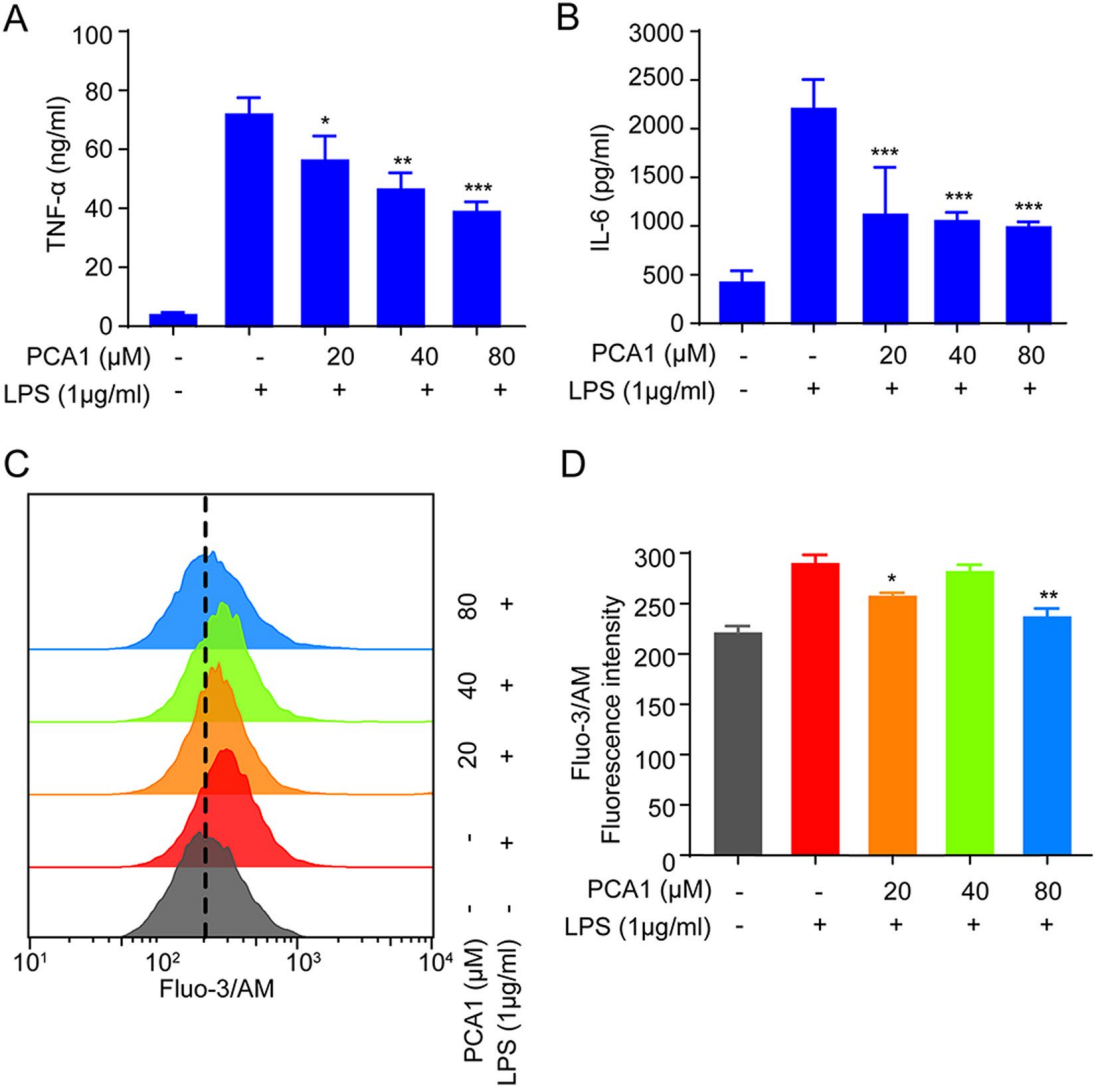
PCA1 reduced LPS-induced pro-inflammatory cytokines in RAW264.7 cells. (**A**,**B**) RAW264.7 cells treated with PCA1 (0, 20, 40, 80 μM) for 1 h and then induced with LPS (1 μg/ml) for 18 h. The TNF-α and IL-6 level was detected using ELISA kits. (**C**) RAW264.7 cells were pretreated with PCA1 (0, 20, 40, 80 μM) for 1 h before co-incubation with LPS (1 μg/ml) for 8 h. Then the cells were marked with Fluo-3/AM for 1 h and assessed by flow cytometry. (**D**) Statistical analysis of the NO per group. *p < 0.05,**p < 0.01, and ***p < 0.001 versus the LPS group.

According to previous study, RAW264.7 cells release large amounts of pro-inflammatory cytokines such as TNF-α, IL-6, IL-1β, *etc*. due to LPS-mediated the inflammatory reaction^20^. Ca^2+^, another inflammatory signal, will lead to influx in cell during inflammatory process^21,22^. Therefore, we explored whether PCA1 could exhibit inflammatory effect on the release of pro-inflammatory cytokines. Our ELISA assay results indicated that PCA1 attenuated TNF-α and IL-6 levels in a dose-dependent manner (Fig. 2A,B). PCA1 can inhibit TNF-α and IL-6 more than 50% under the concentration of 40 μM. Calcium production was determined by Fluo-3/AM (1 μM) for 1 h. Our results showed that 20 μM administration with PCA1 decreased calcium production significantly compared with the control group (Fig. 2C,D). Above all, these findings showed that PCA1 diminished inflammatory responses in LPS-stimulated RAW264.7 cells.

### PCA1 disrupted LPS-induced NF-κB nuclear translocation

**Figure 3:**
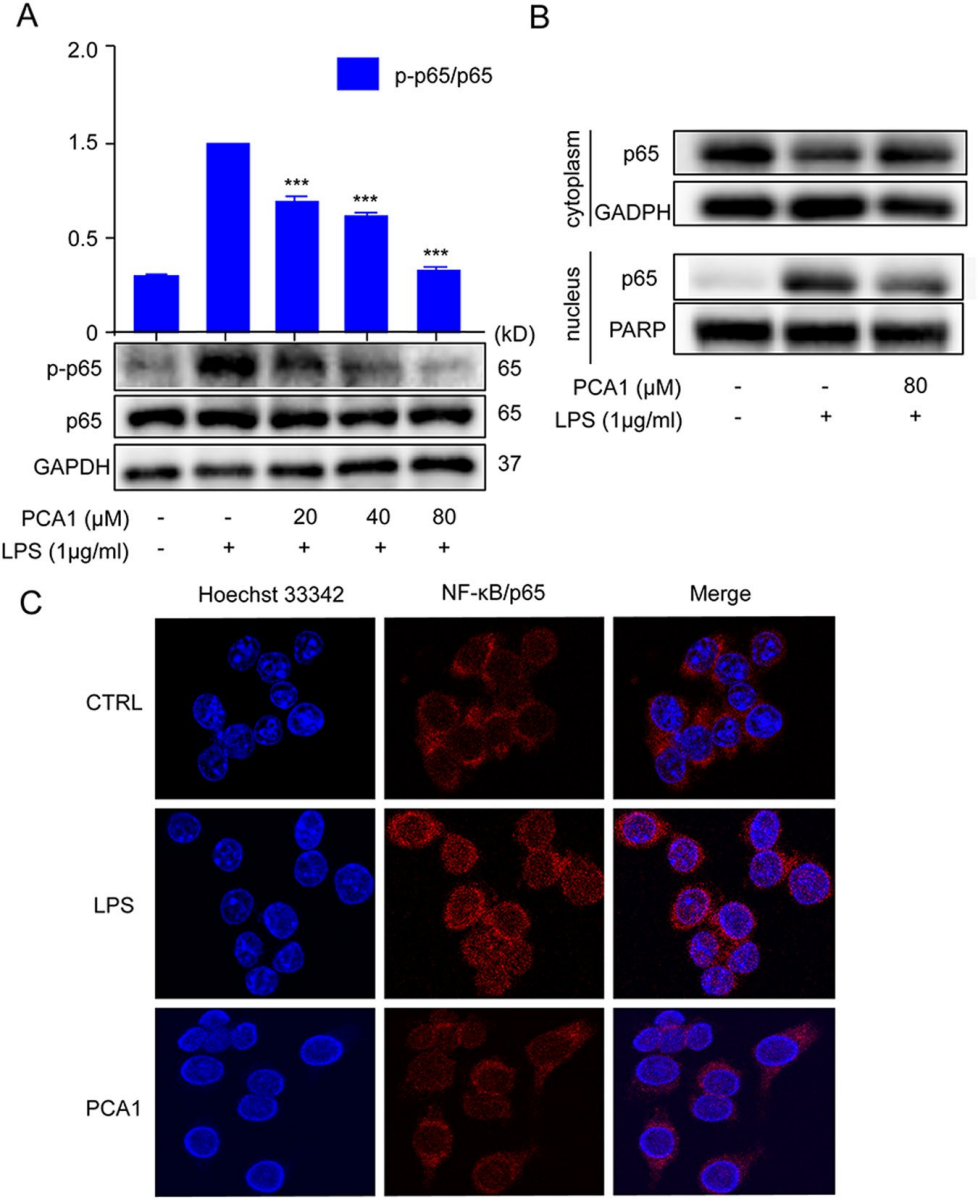
PCA1 hindered LPS-induced NF-κB nuclear translocation. (**A**) RAW264.7 cells treated with PCA1 for 2 h were induced by LPS (1 μg/ml) for 2 h. p-p65 and p65 protein level was detected by Western blotting. (**B**) RAW264.7 cells were pretreated with PCA1 (80 μM) for 1 h and then stimulated with LPS (1 μg/ml) for 1 h. The localization of p65 in the cytoplasm and nucleus were detected by Western blotting. (**C**) RAW264.7 cells treated with for PCA1 for 2 h were included with LPS for 2 h. p65 translocation was determined using immunofluorescence analysis. The grouping of gels/blots cropped from different parts of the same gel (targets vs loading control) or different gels (phosphorylation). The full blots are shown in Supplementary Information. ***p < 0.001 versus the LPS group.

There has been reported that LPS can induce translocation of NF-κB/p65 from the cytoplasm to the nucleus^23^. And the nucleus translocation of NF-κB can regulate the release of large amounts of inflammatory mediators such as TNF-α, IL-6, IL-1β, NO, and iNOS^24^. As shown in Fig. 3A, we found that LPS-induced RAW264.7 cells increased p-p65 protein expression, while the pre-treatment with PCA1 significantly attenuated p-p65 protein expression. Furthermore, LPS promoted NF-κB nucleus translocation. However, PCA1 also prevented the nucleus translocation of NF-κB with the concentration of 80 μM (Fig. 3B). Meanwhile, our immunofluorescence results showed that LPS increased NF-κB nucleus translocation, which was decreased by PCA1 (Fig. 3C). Based on the results above, we have a conclusion that PCA1 suppressed NF-κB nuclear translocation.

### The effect of PCA1 on NF-κB and MAPKs pathways in RAW264.7 cells

**Figure 4:**
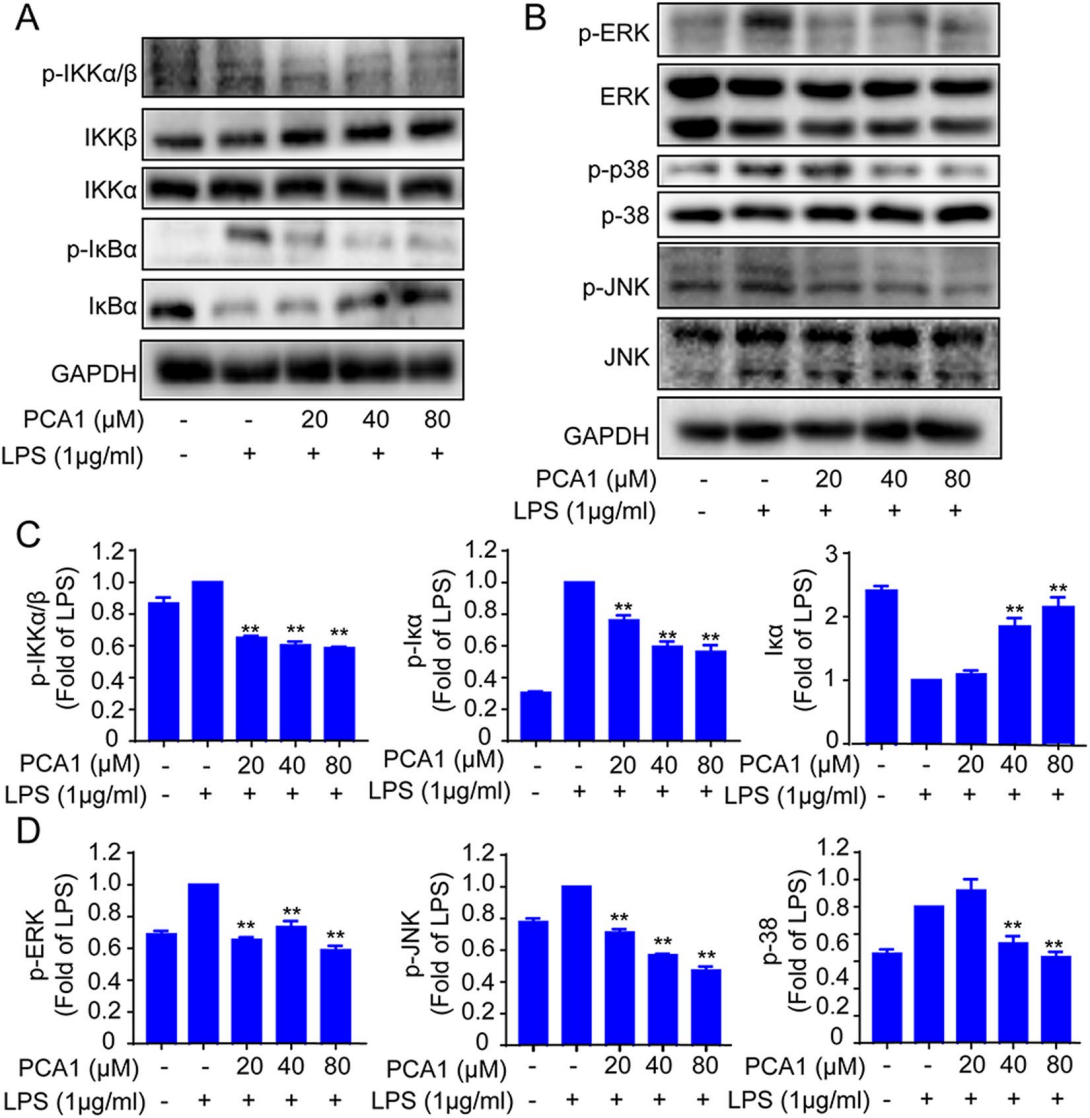
Impact of PCA1 on the NF-κB and MAPK pathway in RAW264.7 cells. (**A**,**B**) RAW264.7 cells treated with PCA1 for 2 h were induced by LPS. The NF-κB and MAPKs signaling pathway expression proteins were detected using Western blotting. (**C**,**D**) Statistical analysis of the IκB-α, IKK-α/β, IKK-α, IKK-β, p-JNK1/2, JNK1/2, p-ERK1/2, ERK1/2, p-p38, and p38 per group. The grouping of gels/blots cropped from different parts of the same gel (targets vs loading control) or different gels (phosphorylation). The full blots are shown in Supplementary Information. *p < 0.05, **p < 0.01, and ***p < 0.001 versus the LPS group.

PCA1 plays a vital role in NF-κB pathway, which remarkably inhibited NF-κB activity and translocation^25^. As shown in Fig. 4A,C, the phosphorylation of IκBα induced by LPS was inhibited, while IκBα protein level was increased by pretreatment with PCA1 in a dose-dependent manner in RAW264.7 cells. Then PCA1 also inhibited IKKα/β phosphorylation induced by LPS with no impact of the expression of IKKα and IKKβ.

The proteins of MAPKs family (JNK1/2, ERK1/2, and p38MAPK) involved in anti-inflammatory responses, also played an important role in inflammatory diseases^26^. As shown in Fig. 4B,D, PCA1 remarkably suppressed the protein level of p-JNK1/2, p-ERK1/2, and p-p38MAPK with no impact on the expression of total JNK1/2, ERK1/2, and p38MAPK. Collectively, this study demonstrated that PCA1 exhibited anti-inflammatory activity through NF-κB and MAPKs signaling pathway.

### PCA1 inhibited ROS production and reduced mitochondrial membrane potential (MMP)

**Figure 5:**
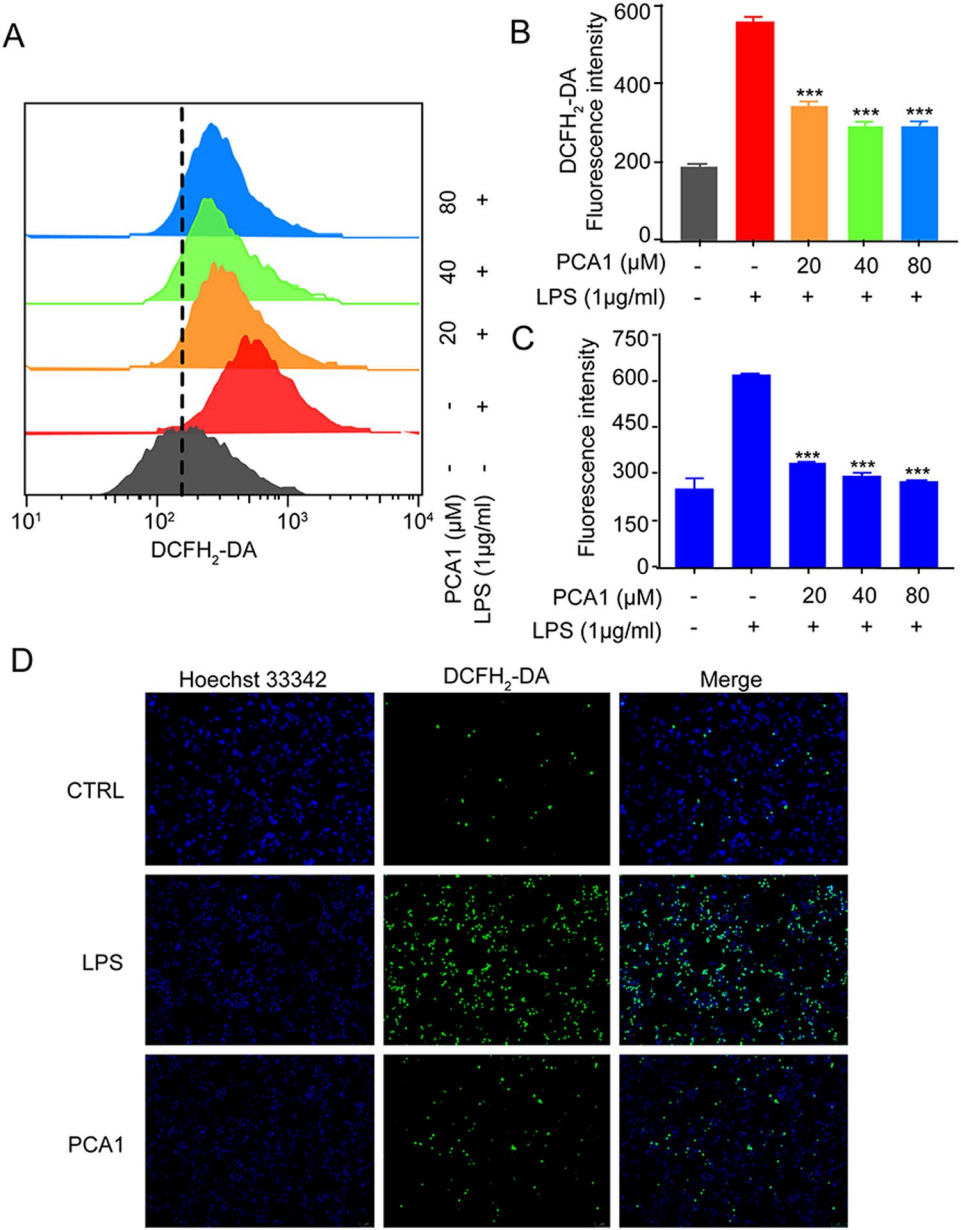
PCA1 inhibited ROS generation. (**A**) RAW264.7 cells treated with for PCA1 (20, 40, 80 μM) for 1 h were incubated with LPS for 8 h. Cells were stained with DCFH_2_-DA (1 μM) for 30 min. The fluorescence intensity was determined by flow cytometry at the FITC channel. (**B**) Statistical analysis of the ROS per group. (**C**) RAW264.7 cells were seeded into 96-well plates and cultured overnight. Cells pretreated with PCA1 (0, 20, 40, 80 μM) for 1 h were stimulated with LPS (1 μg/ml) for 8 h. The ROS level was determined by a ROS kit (Sigma MAK143) according to the manufacturer’s instructions. (**D**) ROS level in cells was stained with DCFH_2_-DA. The images were captured by fluorescence microscopy. ***p < 0.001 versus the LPS group.

**Figure 6:**
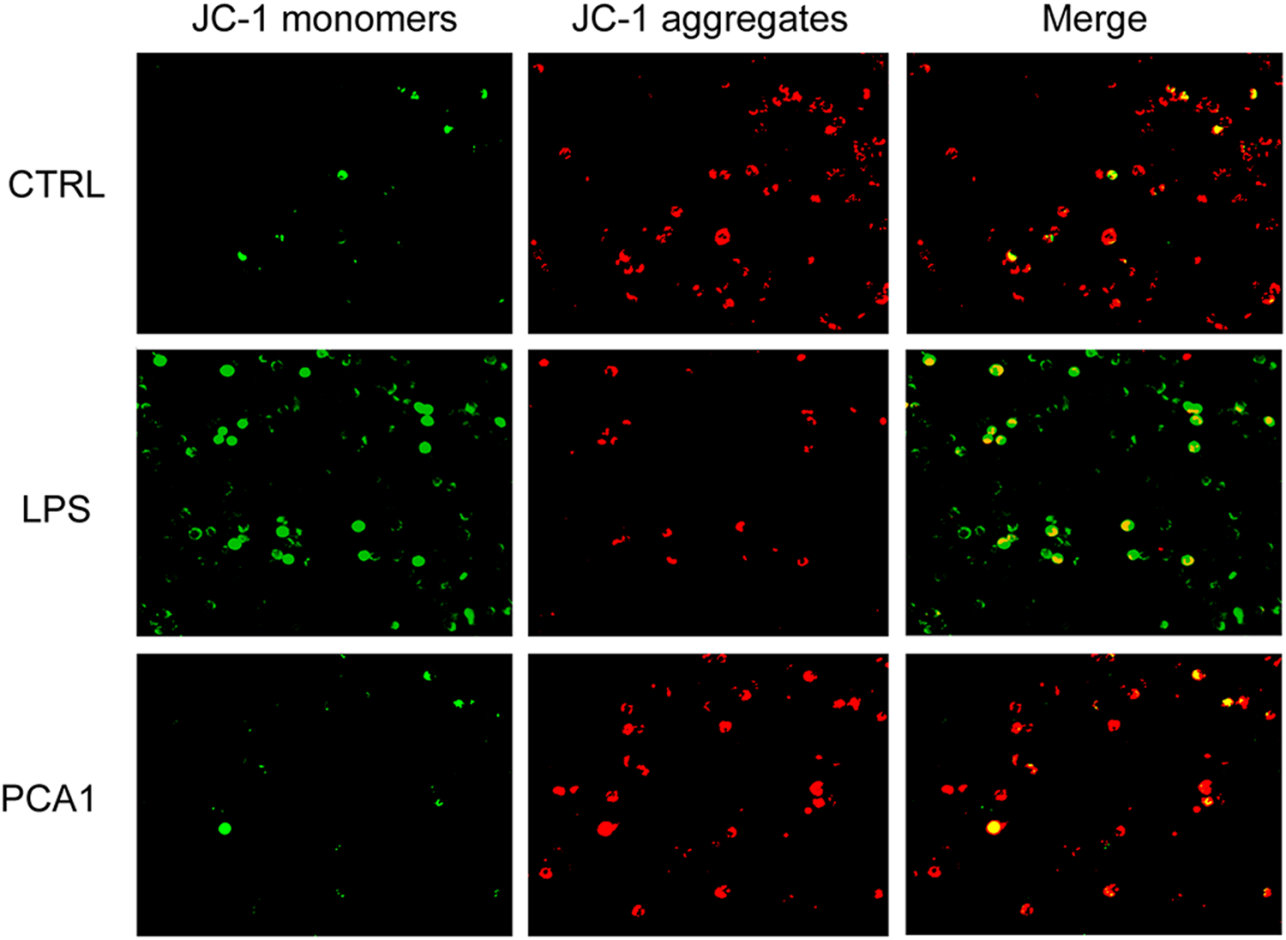
PCA1 reduced mitochondrial membrane potential (MMP). RAW264.7 cells treated with for PCA1 for 1 h were incubated with LPS for 8 h. The probe of JC-1 (5 μg/mL) was employed to detect cells for 20 min. Images were captured by fluorescence microscopy.

ROS, a vital signaling factor, plays a critical role in inflammatory disorder. An increase of ROS level can be induced by LPS, meanwhile, inflammation can also cause accumulation of ROS to further promote oxidative stress^27^. In our study, ROS generation was examined by DCFH_2_-DA. As shown in Fig. 5, PCA1 significantly suppressed LPS-induced ROS generation in RAW264.7 cells. In addition to the flow cytometry assay, immunofluorescent assay and intracellular ROS kit were employed to detect the intracellular ROS generation. The results indicated that PCA1 decreased intracellular ROS level (Fig. 5A–D). LPS disrupts the stability of mitochondrial membrane potential (MMP), which is not conducive to maintaining normal physiological functions of cells^28^. As shown in Fig. 6, the JC-1 monomer increased by LPS-stimulation (green staining), but the pretreatment with PCA1 decreased the JC-1 monomer and restored it to aggregates (red fluorescence). Taken together, PCA1 inhibited ROS production and aggrandized MMP in RAW264,7 cells.

### Impact of PCA1 on Keap1-Nrf2 signaling pathway in RAW264.7 cells

**Figure 7:**
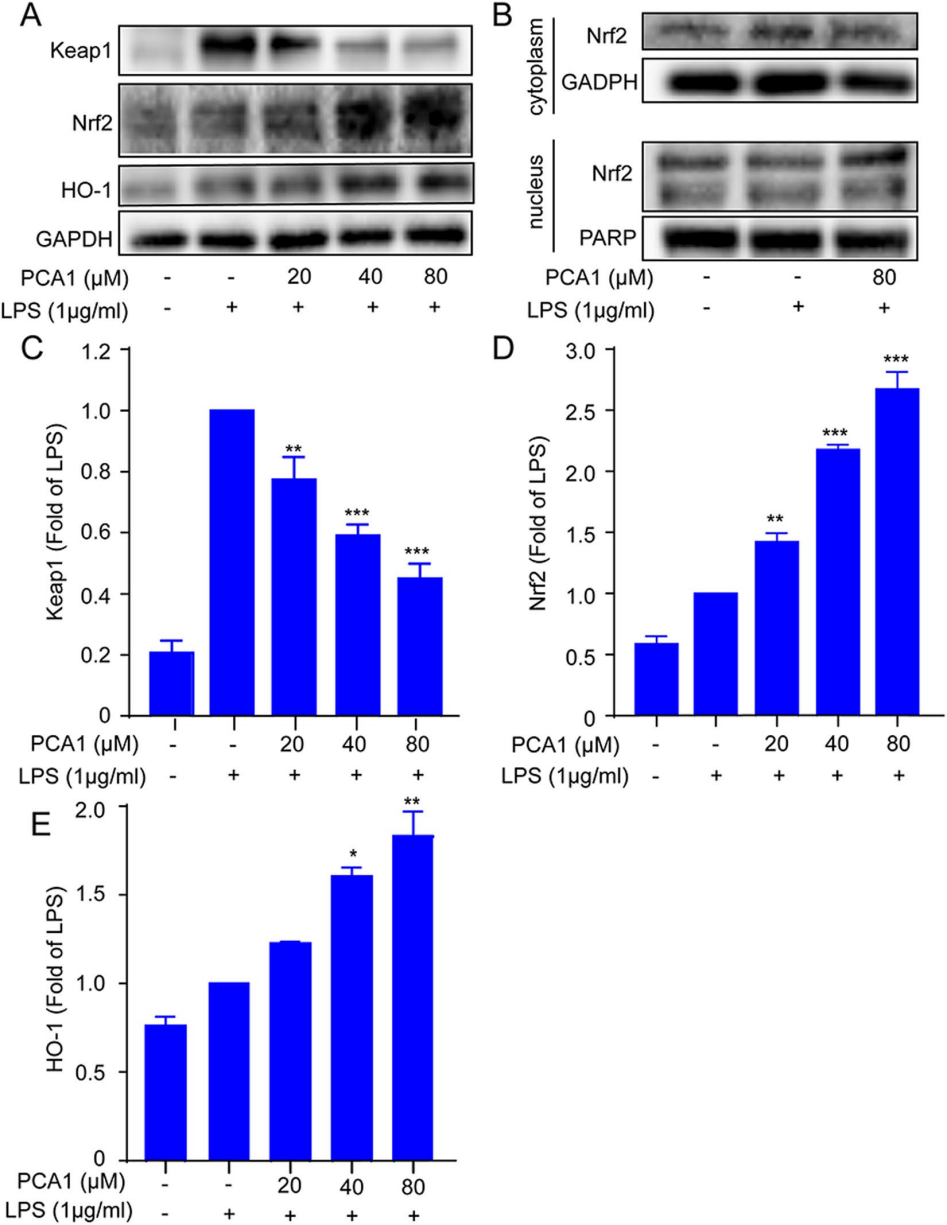
Impact of PCA1 on Keap1-Nrf2 signaling pathway in RAW264.7 cells. (**A**) RAW264.7 cells treated with for PCA1 for 1 h were incubated with LPS for 8 h. the expression of Keap1, Nrf2, and HO-1 were detected using Western blotting. (**B**) RAW264.7 cells treated with for PCA1 for 2 h were co-cultured with LPS for 2 h. The protein expression of Nrf2 in cytoplasm and nucleus were detected by Western blotting. (**C**–**E**) Statistical analysis of the Keap1, Nrf2, and HO-1 content per group. The grouping of gels/blots cropped from different parts of the same gel (targets vs loading control) or different gels (phosphorylation). The full blots are shown in Supplementary Information. *p < 0.05, **p < 0.01, and ***p < 0.001 versus the LPS induced group.

The recent study demonstrated that LPS activates the Nrf2 signaling pathway in RWA264.7^29,30^. In the study, the Nrf2 pathway was investigated. As shown in Fig. 7A,C,D, LPS stimulation induced upregulation of Keap1 expression, while pretreatment with PCA1 slightly downregulated Keap1 protein expression. Pretreatment with PCA1 increased Nrf2 and HO-1 protein expressions in RWA264.7 cell. The pretreatment with PCA1 increased Nrf2 protein level in the nucleus but decreased the protein level in the cytoplasm, compared with NC and LPS stimulation group (Fig. 7B).

### Toll-like receptor participated in PCA1’s anti-inflammatory process

**Figure 8:**
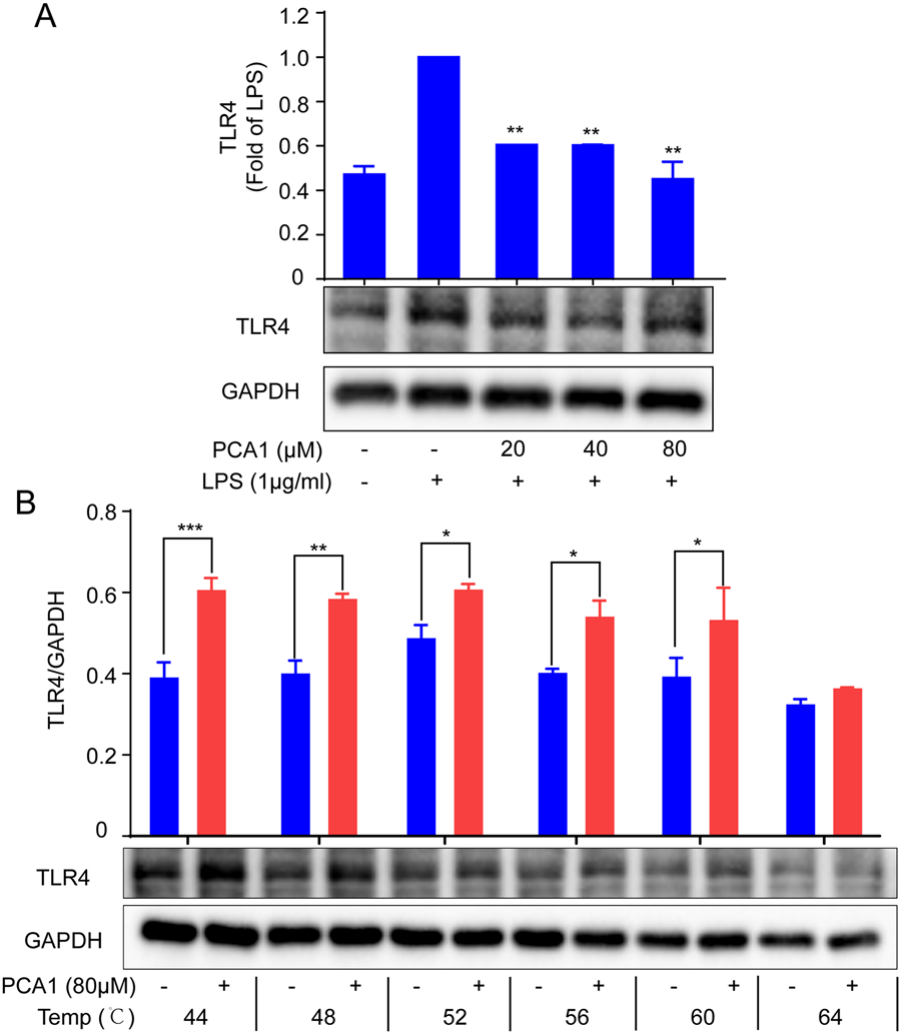
PCA1 binds to TLR4. (**A**) RAW264.7 cells treated with for PCA1 (0, 20, 40, 80 μM) for 2 h were incubated with LPS for 2 h. The expression of TLR4 were detected using Western blotting. (**B**) Treated with PCA1 (80 μM) for 2 h, cells were lysed by RIPA and total proteins were collected. Then, proteins were equally divided into 6 parts, which were heated at 44, 48, 52, 56, 60, and 64 °C for 3 min, respectively. The corresponding loading buffer were added into the proteins, which were analysed by western blotting. The grouping of gels/blots cropped from different parts of the same gel (targets vs loading control). The full blots are shown in Supplementary Information. *p < 0.05, **p < 0.01, and ***p < 0.001 versus the LPS-induced/compared group.

TLR4 that is specifically found on multiple cells makes an important difference in the inflammatory process^31^. In presence of LPS and MD2, TLR4 dimerizes, modulating the NF-κB, MAPK, and other signaling cascades, eliciting pathogen-specific innate immune responses via pro-inflammatory cytokine release^23^. To evaluate the PCA1’s effect on TLR4, the cellular thermal shift assay was employed to detect whether PCA1 binds to TLR4. Our results indicated that PCA1 attenuated LPS-induced TLR4 expression and PCA1 bond to TLR4 (Fig. 8A,B).

## Discussion

The inflammatory reaction is a common pathological reaction in life, which exists in various tissues and organs^32^. Inflammation is related to the occurrence and development process of many diseases including stroke, arthritis, neurodegenerative disease, and cardiovascular disease^33^. Therefore, the inhibition of inflammation plays a key role in inflammation-related diseases therapy. In previous studies, we found that PCA1 is one of the monomers isolated from procyanidins, which could reduce the inflammatory response^34^. This study aimed to elucidate the molecular mechanism of the anti-inflammatory effect of PCA1.

Macrophages play vital roles in regulating inflammatory responses^25^. LPS, one of the main components of the outer membrane of gram-negative bacteria, can bind to the TLR4 receptor and promote inflammation^35^. Thus, the RAW264.7 cell inflammatory model induced by LPS was used in this study. Under the stimulation of LPS, macrophages can release a variety of inflammatory factors, such as TNF-α, IL-6, NO, and IL-1β^36^. Therefore, blocking the occurrence of these inflammatory mediators in inflammatory responses may inhibit the development of inflammation. In our study, PCA1 attenuated NO, iNOS, TNF-α, and IL-6 production, but no effect on COX-2 level (Figs 1 and 2). These results suggested that PCA1 has an anti-inflammatory activity.

Calcium plays a crucial role in the stimulated macrophages, which is considered as a second signal messenger on multiple signaling pathways^37^. Stimulation of RAW264.7 cells by LPS leads to an increase level of intracellular calcium. Previous studies have shown that calcium are involved in the transcriptional activation of inflammatory cytokines and chemokines besides IL-6, TNF-α, IL-1β, and NO^38^. Our study illustrated that PCA1 decreased the production of calcium (Fig. 3).

Abundant evidence has shown that LPs binding with TLR4 can activate many inflammatory pathways, such as NF-κB pathway and MAPKs pathaway^39^. NF-κB signaling pathway plays a necessary role in the pathological process of the inflammatory response. NF-κB is a multipotent transcription factor of the protein family. It can promote transcriptional expression through binding to κB DNA elements^40^. Normally, NF-κB and IκB binds together and forms a complex in the cytoplasm. However, provoking the degradation of IκB and nucleus translocation of NF-κB subunit p65 from the cytoplasm is induced by LPS stimulation of inflammation^41^. The upregulation of p65 in the nuclear promotes the release of a large number of cytokines and chemokines^10^. Our data indicated that PCA1 dramatically attenuated LPS-mediated upregulation of phosphorylation of IKKα/β, IκB and p65, degradation of IκB, and the translocation of p65 from the cytoplasm and into the nucleus (Fig. 3). It is reported that TLR4 and MAPKs signaling pathway are also actively involved in inflammatory responses^42^. Enhancing the phosphorylation of JNK1/2, P38, ERK1/2 in LPS-induced RAW264.7 cells^43^. We have shown that PCA1 markedly inhibited the phosphorylation of JNK1/2, P38, ERK1/2 without changing the activation of total JNK1/2, P38, ERK1/2 protein level (Fig. 4). In addition, our results showed that PCA1 binds to TLR4 (Fig. 8). Taken collectively, PCA1 exhibited anti-inflammatory response by mediating NF-κB and MAPKs pathways.

ROS generation mediates a variety of inflammatory signaling pathways which promotes inflammation reaction^44^. MMP is also an important inflammatory response signal as well as ROS^45^. These suggested that either the inhibition of ROS or the promotion of MMP can also be the most important therapeutic target of inflammatory diseases^46^. In this study, PCA1 significantly reduced ROS production and restored mitochondrial membrane potential (Figs 5 and 6).

HO-1, an antioxidant enzyme in the stress response, catalyzing the degradation of heme, biliverdin, and carbon monoxide (CO), also plays an important role in inflammation and oxidation responses^47^. Chronic inflammation has been reported in HO-1 deficient models^48^. As the main protein regulating HO-1 expression, Nrf2 is distributed in the cytoplasm together with Keap1 normally. However, when Keap1 was degraded under oxidative stress, Nrf2 was released and migrated to the nucleus, then induced HO-1 expression^49^. Our results showed that pretreatment with PCA1 decreased Keap1 expression, promoted Nrf2 nuclear translocation and induction of HO-1 *in vitro* (Fig. 7).

**Figure 9:**
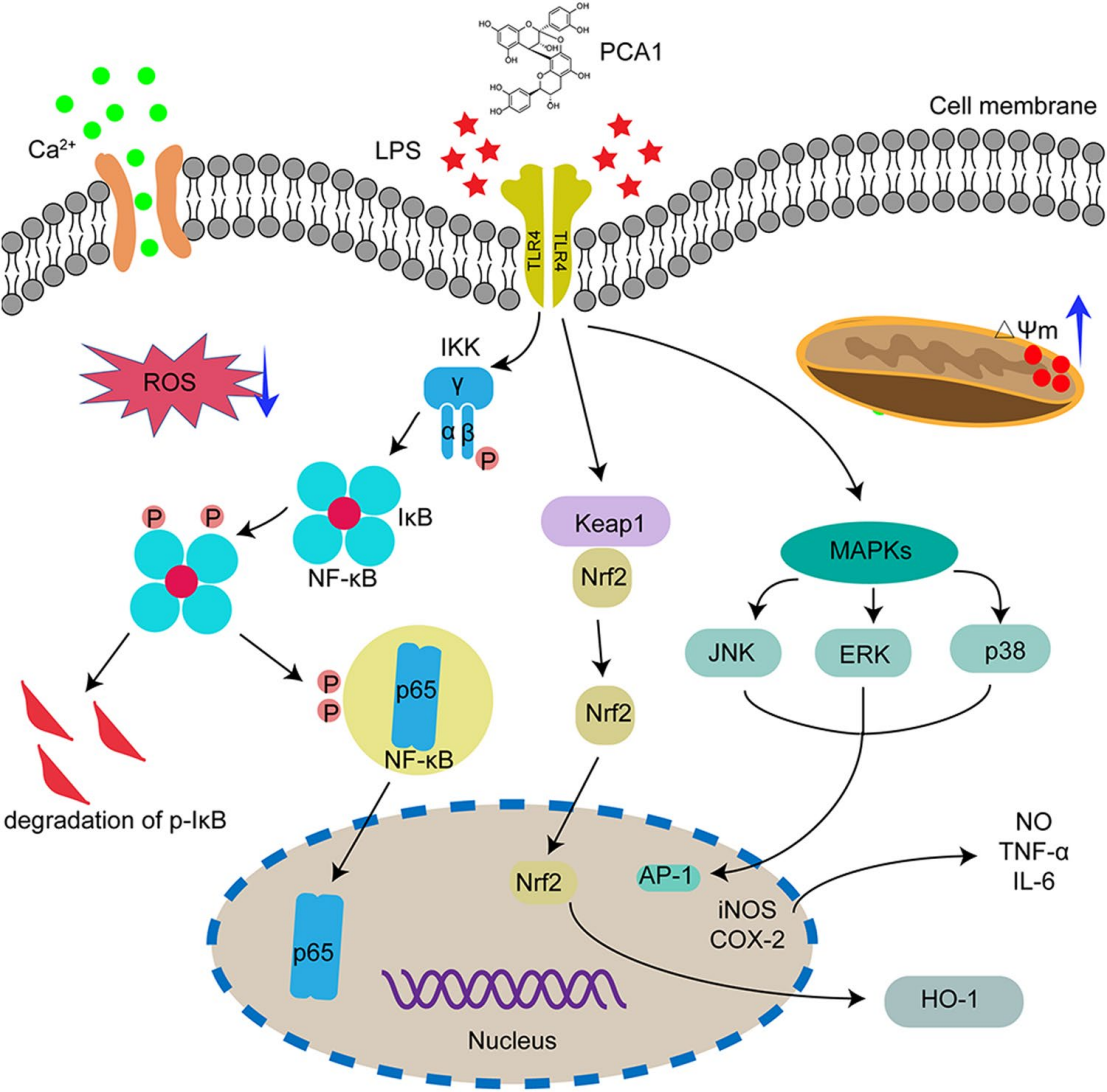
The schematic of the anti-inflammatory activities of procyanidin A1 *in vitro*. Procyanidin A1 (PCA1) exerts anti-inflammatory activity, which decreases calcium exclusion, reactive oxygen species (ROS) generation, and the release of pro-inflammatory cytokines through NF-kB, MAPK, and Nrf2/HO-1pathways. LPS, lipopolysaccharides; TNF-α, tumor necrosis factor-α; NO, nitric oxide; COX-2, cyclooxygenase-2; iNOS, inducible nitric oxide synthase; NF-κB, nuclear factor-κB; IL-6, interleukin-6; MAPK, mitogen-activated protein kinase; JNK, c-Jun N-terminal kinase; ERK, extracellular regulated protein kinase; ROS, reactive oxygen species; Nrf2, nuclear factor erythroid 2-related factor; HO-1, heme oxygenase-1.

In conclusion, we proved that PCA1 exhibited anti-inflammatory and anti-oxidant effects on RAW264.7 cells *via* the NF-κB and MAPK signaling pathway (Fig. 9). Thus, PCA1 is a potential agent for the research and development of the treatment with inflammatory diseases.

## Materials and Methods

### Chemical and reagents

Procyanidin A1 was purchased from PUSH BIO-TECHNOLOGY, (Chengdu, China). Lipopolysaccharides from *Escherichia coli* O111:B4, Griess reagent (modifiziert-G4410), 2′,7′-Dichlorodihydrofluorescein diacetate (DCFH_2_-DA) were obtained from Sigma-Aldrich (St. Louis, MO, USA). Dulbecco’s modified eagle medium (DMEM), Fluo-3/AM, NO detector DAF-FM, fetal bovine serum (FBS) were purchased from Life Technologies/Gibco Laboratories (Grand Island, NY, USA). ELISA kits were obtained from Neobioscience (Shenzhen, China). Antibodies against COX-2 (#4842), NF-κB/p65 (#8242T), p-NF-κB/p65 (#3033), IKKα (#2682), IKKβ (#8943T), p-IKKα/β (#2078), IκBα (#4814), p-IκBα (#2859), JNK1/2 (#9252T), p-JNK1/2 (#4668T), ERK1/2 (#4695T), p-ERK1/2 (#4370T), p38MAPK (#9212), p-p38MAPK (#4511), Keap-1 (#4678), Nrf2 (#12721), HO-1 (#70081), PARP (#9532) and GAPDH (#5174) were purchased from Cell Signaling (Beverly, MA, USA).

### Cell culture

RAW264.7 (murine macrophage) cell line was from the Cell Bank of the Chinese Academy of Sciences (Shanghai, China). The cell line was cultured in DMEM High glucose normal culture medium including 10% FBS, 100 U/mL penicillin and 100 mg/mL streptomycin in a humidified incubator, where containing 5% CO_2_ and maintaining at 37 °C.

### Cell viability assay

Cell viability was measured by MTT assay. Probably 1 × 10^5^ RAW264.7 cells were seeded in 96-well plates and cultured overnight, then treated with PCA1 (0, 20, 40, 80 μM) for 24 h, following MTT (5 mg/ml) was added to each well and cultured for 3 h. Then removing culture media, the crystal violet was dissolved with DMSO (100 μL/well), which was determined at 570 nm using microplate reader^50^.

### Assessment of nitrite level and cytokine release

RAW264.7 cells were seeded in 12-well plates with a density of 2 × 10^5^ cells/well for 24 h. Cells were pretreated with PCA1 for 1 h, and then inducted an inflammatory response by LPS (1 μg/ml) for 18 h. Collection of culture media for the detection of nitrite and inflammatory cytokines with Griess Regent and ELISA kit, respectively.

### Western blotting analysis

RAW264.7 cells were seeded into a dish or 6-well plates and fostered overnight. PCA1 (20, 40, 80 μM) was pretreated at indicated time points, after LPS induction for a certain period time. Total cell proteins were extracted using RIPA (1% PMSF and 1% cocktail). According to the manufacturer’s instruction, the extraction of cytoplasmic and nuclear protein with the kit (Beyond time, Shanghai, China) was used to obtain the cytoplasmic and nuclear proteins. BCA protein kit (Waltham, MA, USA) was employed to determine protein concentrations. The denatured protein was separated by 8% or 10% SDS-PAGE gels and transferred them to PVDF membrane (Millipore, Billerica, MA, USA). After blocking the PVDF membrane with 5% nonfat milk for 1 h, the PVDF membrane was incubated with primary antibodies (1:1000) for more than 12 h at 4 °C. After washing with TBST and incubated with secondary antibody (1:5000) for 2 h at room temperature, the membrane was exposure by ChemiDoc™ MP Imaging System (Bio-Rad, Hercules, CA, USA).GAPDH was used as a housekeeping protein.

### Flow cytometry assay

RAW264.7 cells were seeded in 24-well plates with a density of 1.5 × 10^5^ cells per well and cultured overnight. The Cells were pretreated with PCA1 for 1 h, and then treated with or without LPS (1 μg/ml). Different probes were used to detect the corresponding indicators, including NO detector DAF-FM (5 μM, 1 h)^51^, ROS detector DCFH_2_-DA (10 μM, 30 min), Ca^2+^ detector Fluo-3/AM (1 μM, 1 h) or MMP detector JC-1 (10 μg/ml, 30 min). After probes incubation, cells were collected and tested by flow cytometry (Becton-Dickinson, Franklin Lakes, NJ, USA).

### Immunofluorescence assay

RAW264.7 cells were seeded in confocal dishes with a density of 15 × 10^5^ cells/ml overnight. Then these cells were pretreated with PCA1 (80 μM) for 4 h and LPS-stimulated (1 μg/ml) for 2 h. After fixed, punched, blocked, the cells in the dished were incubated with NF-κB p65 antibody (1:100) overnight at 4 °C. Finally, cells were incubated with Alexa Fluor 594 secondary antibody for 1 h. Nuclei were revealed by Hoechst 33,342 staining. Fluorescence images were collected under a confocal microscope system (Leica, Wetzlar, Germany).

### Fluorescence assay

RAW264.7 cells were seeded in 96-well plates with a density of 4 × 10^5^ cells/ml overnight. Cells were pretreated with PCA1 (80 μM) for 1 h and then incubated with or without LPS for another 8 h. Staining with JC-1 (10 μg/ml) and DCFH_2_-DA (100 μM) for 30 min. Fluorescence images were captured by fluorescence microscopy.

#### Cellular thermal shift assay

RAW264.7 cells were seeded into a dish (2 × 10^6^ cells) overnight. Treated with PCA1 (80 μM) for 2 h, cells were lysed by RIPA and total proteins were collected. Then, proteins were equally divided into 6 parts, which were heated at 44, 48, 52, 56, 60, and 64 °C for 3 min, respectively. The corresponding loading buffer were added into the proteins, which were analysed by western blotting.

### Statistical analysis

Date are presented as means ± SD or means ± S.E.M. All experiments are repeated at least three times. Data are normally distributed and analyzed by one-way-ANOVA or Student’s *t*-test by GraphPad Prism 6.0 software. Results are considered as statistical significance when *p* < 0.05.

## Supplementary information

Supplementary information
