# Supplementary material for: Procyanidin A1 Alleviates Inflammatory Response induced by LPS through NF-κB, MAPK, and Nrf2/HO-1 Pathways in RAW264.7 cells
Source: Sci Rep. 2019 Oct 21;9:15087. doi: 10.1038/s41598-019-51614-x (PMC6803657; doi:10.1038/s41598-019-51614-x)
Supplement: Supplementary file 1 — Supplementary information [file 41598_2019_51614_MOESM1_ESM.pdf]

Procyanidin A1 Alleviates Inflammatory Response induced by LPS through NF-κB, MAPK, and Nrf2/HO-1 Pathways in RAW264.7 cells

Shan Han[1,2#], Hongwei Gao[1,2#], Shaoru Chen[3], Qinqin Wang[1,2], Xinxing Li[1,2], Li-Jun Du[4], Jun Li[5], Ying-Ying Luo[5], Jun-Xiu Li[1,2], Li-Chun Zhao[1,2*], Jianfang Feng[1,2*], Shilin Yang[1,2]

[1] College of Pharmacy, Guangxi University of Chinese Medicine, Nanning 530000, China; [2] Guangxi Engineering Technology Research Center of Advantage Chinese Patent Drug and Ethnic Drug Development, Nanning, 530020; [3] Department of Integrative Medical Sciences, Northeast Ohio Medical University, Rootstown, Ohio 44272, USA [4] School of Life Sciences, Tsinghua University, Beijing 100084, China; [5]State Key Laboratory of Innovative Drug and Efficient Energy-Saving Pharmaceutical Equipment, Jiangxi University of Traditional Chinese Medicine, Nanchang 330004, China;

Running Title: Procyanidin A1 shows anti-inflammatory activity

# Shan Han and Hongwei Gao equally contributed to this work.

[*]Co-corresponding Authors:

Dr. Li-Chun Zhao; Email: hyzlc@126.com; Address: College of Pharmacy, Guangxi University of Chinese Medicine, Nanning 530000, China; Tel: +86-771-4953513 Fax: +86-771-4953513

Dr. Jian-Fang Feng; Email:fengjianfang@vip.163.com; Address: College of Pharmacy, Guangxi University of Chinese Medicine, Nanning 530000, China; Tel: +86-771-4953513 Fax: +86-771-4953513

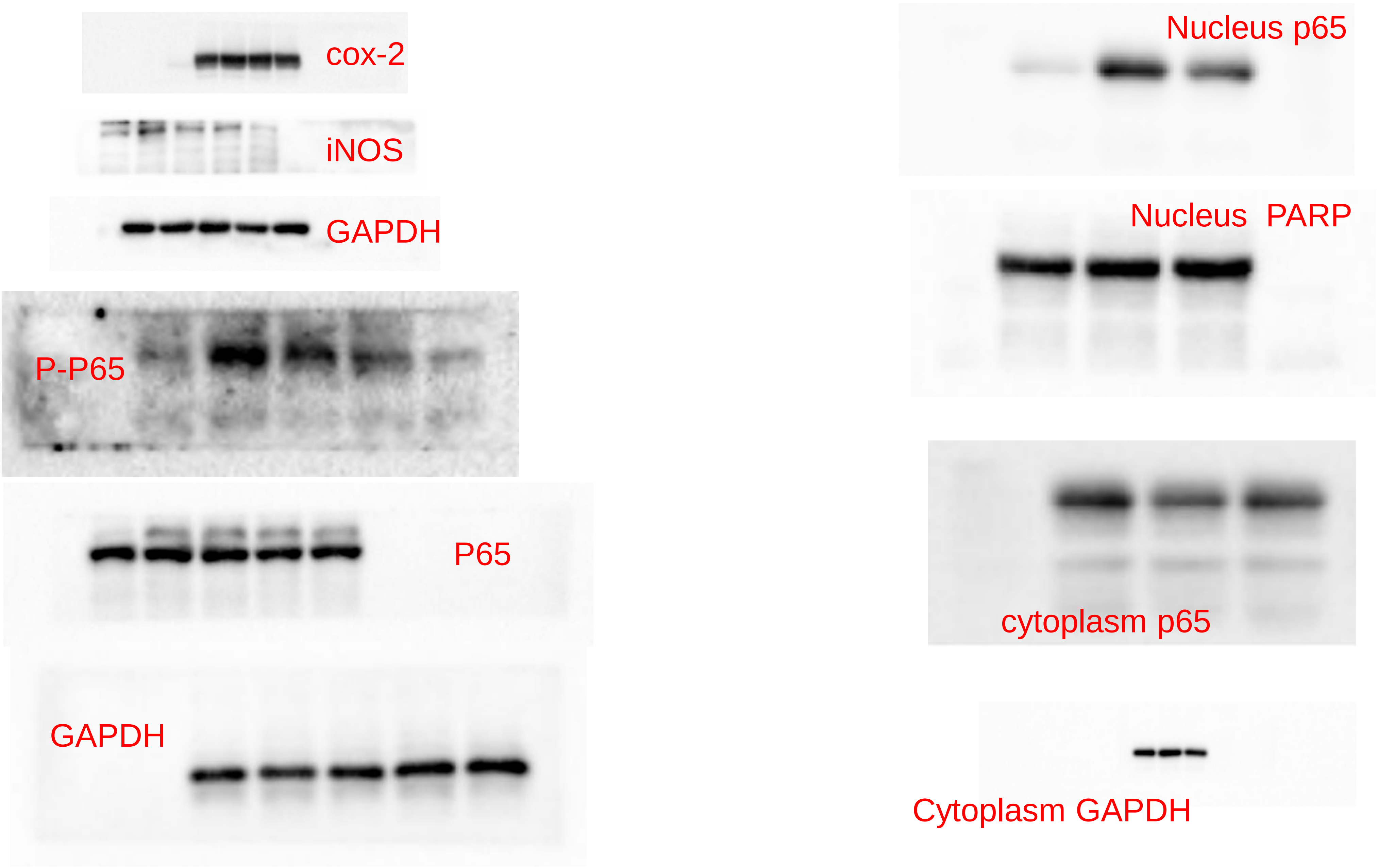
cox-2
iNOS
GAPDH
P-P65
P65
GAPDH
Nucleus p65
Nucleus PARP
cytoplasm p65
Cytoplasm GAPDH

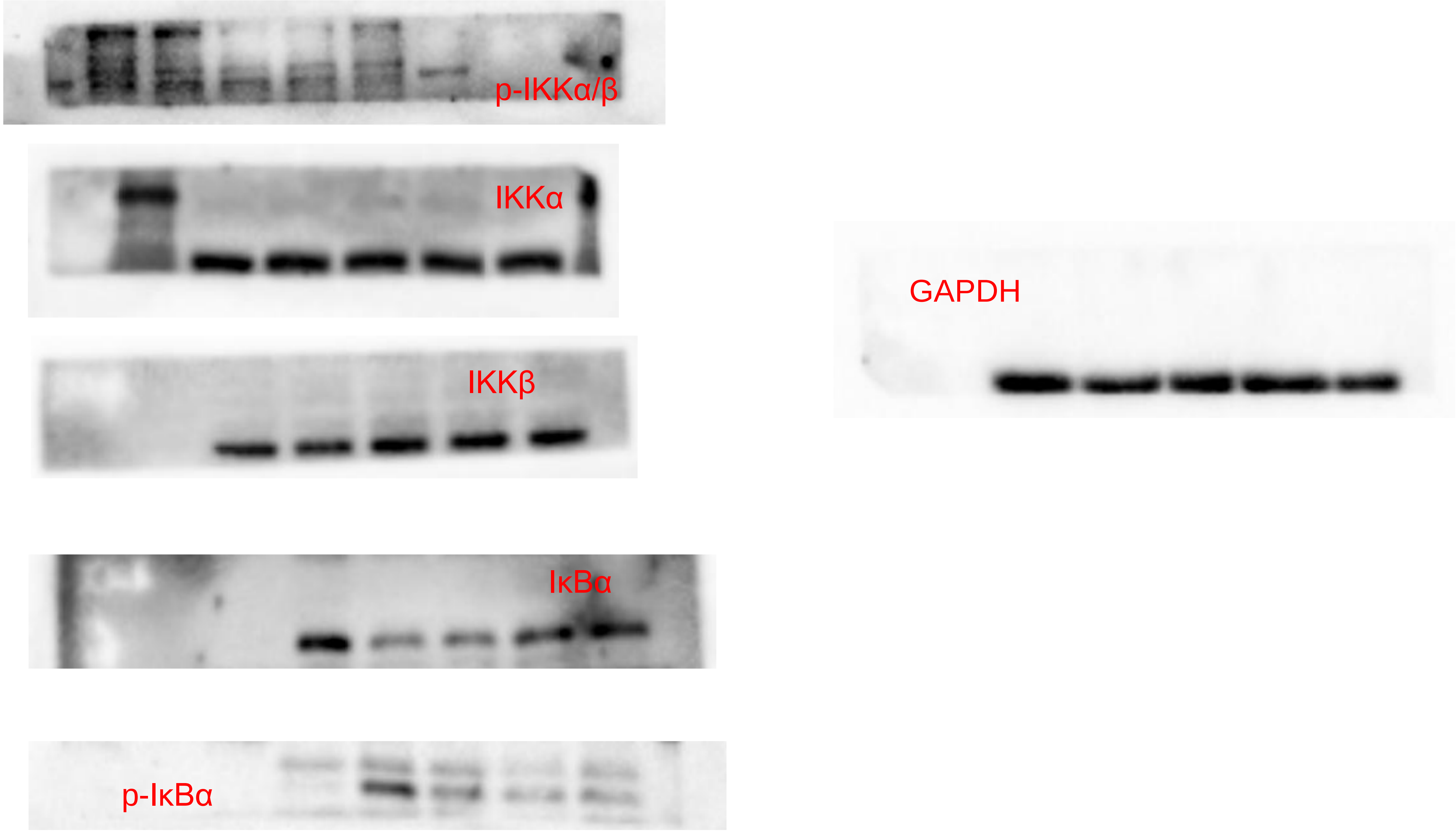

p-IKKα/β
IKKα
IKKβ
IκBα
p-IκBα
GAPDH

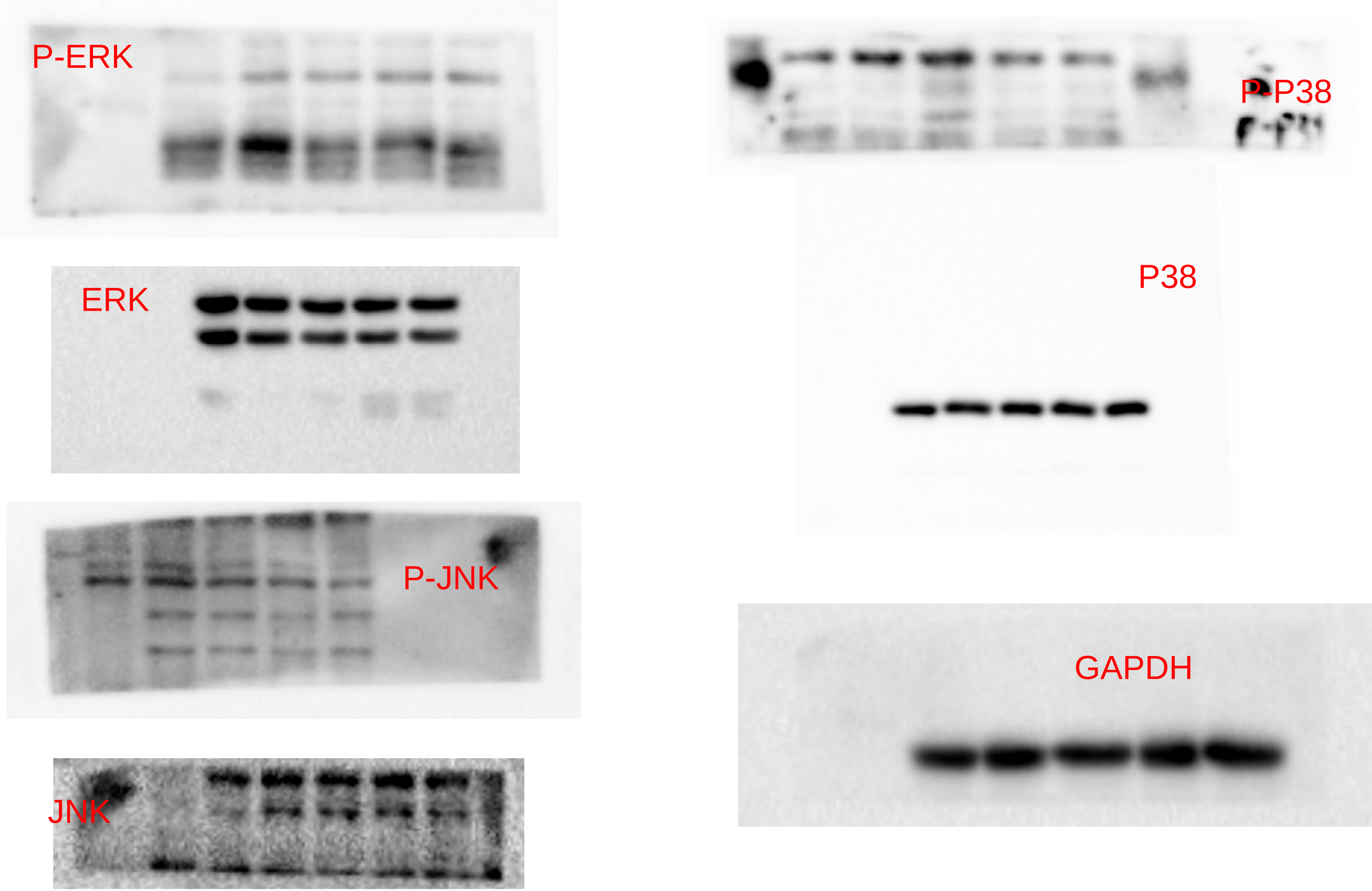
P-ERK
ERK
P-JNK
JNK
P-P38
P38
GAPDH

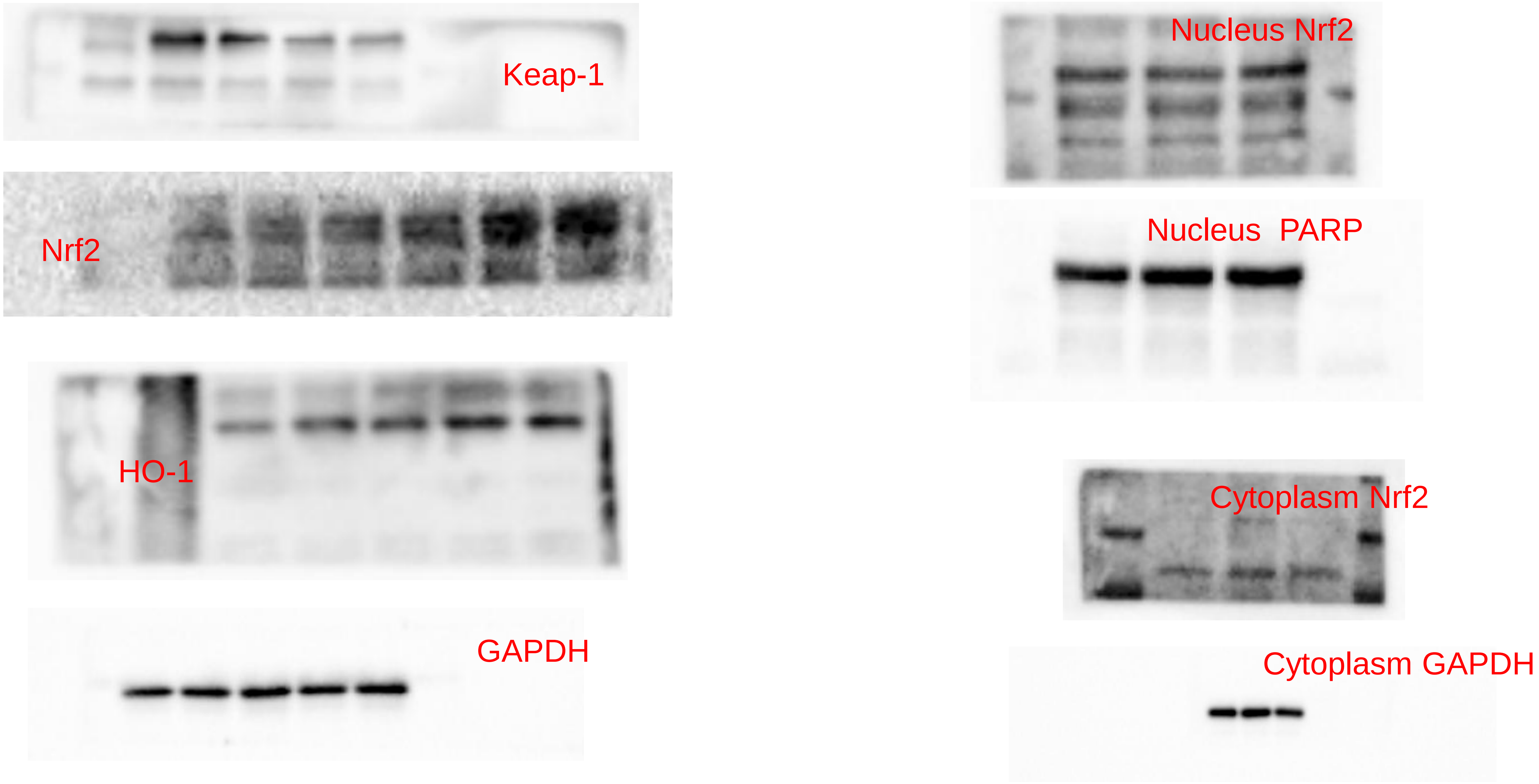
Keap-1
Nrf2
HO-1
GAPDH
Nucleus Nrf2
Nucleus PARP
Cytoplasm Nrf2
Cytoplasm GAPDH

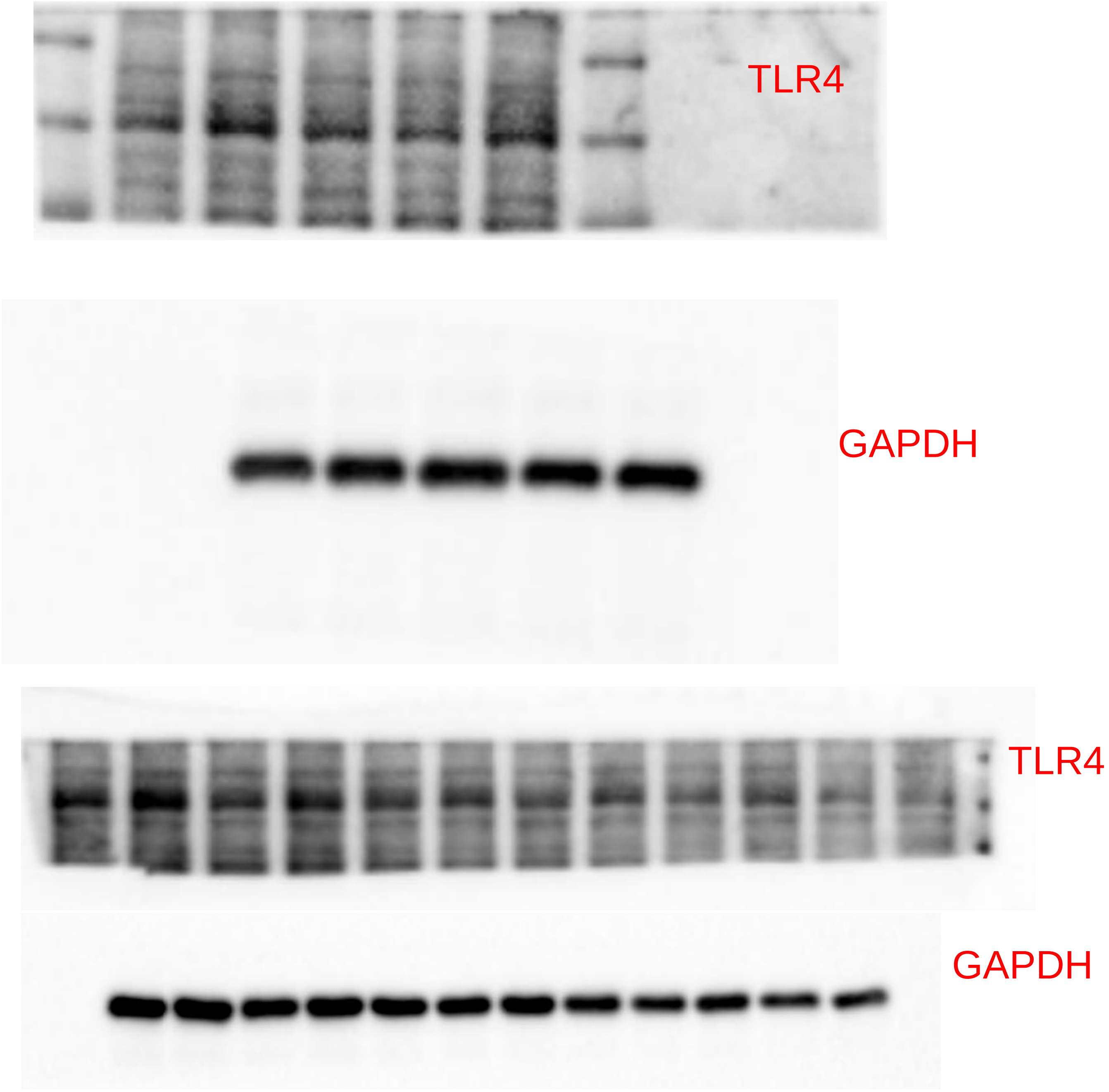
TLR4
GAPDH
TLR4
GAPDH
